# Setting Up a Haplobank: Issues and Solutions

**DOI:** 10.1007/s40778-015-0011-7

**Published:** 2015-04-17

**Authors:** Jacqueline Barry, Johan Hyllner, Glyn Stacey, Craig J. Taylor, Marc Turner

**Affiliations:** 1 Cell Therapy Catapult, 12th Floor Tower Wing, Guy’s Hospital, Great Maze Pond, London, SE1 9RT UK; 2 Division of Biotechnology/IFM, Linköping University, Linköping, Sweden; 3 National Institute of Biological Standards and Controls, Blanche Lane, South Mimms, Potters Bar, Hertfordshire EN6 3QG UK; 4 Histocompatibility and Immunogenetics (Tissue Typing) Laboratory (Box 209), Cambridge University Hospitals NHS Foundation Trust, Addenbrooke’s Hospital, Hills Road, Cambridge, CB2 0QQ UK; 5 Scottish National Blood Transfusion Service, 21 Ellen’s Glen Road, Edinburgh, EH17 7QT Scotland UK; 6 Scottish Centre for Regenerative Medicine, University of Edinburgh, Edinburgh, Scotland UK

**Keywords:** Good manufacturing practice, Induced pluripotent stem cells, HLA matching

## Abstract

The development of induced pluripotent stem cells offers the possibility of the scalable manufacture of cellular therapies for regenerative medicine. Moreover, donors can be selected on the basis of major transplant antigen systems to match the widest possible number of recipients worldwide, reducing the likely risk of immunological rejection and the degree of immune suppression or tolerance required. If such cell lines are to be broadly available, there will need to be mutual recognition of common standards across different jurisdictions. Extensive international collaboration will be required around issues such as determination of the optimal homozygous human leukocyte antigens (HLA) panel, donor selection, screening and consent, good manufacturing practice (GMP), standards and quality control and regulatory legislation. The challenges in establishing a global GMP induced pluripotent stem cell (iPSC) haplobank are formidable. We argue that now is the time to attempt to reach international agreement around common standards for GMP iPSC manufacture before the field develops in a fragmented manner.

## Introduction

The development of pluripotent stem cells has raised the prospect of the scalable manufacture of cellular therapeutics for a wide range of the degenerative disorders, which currently afflict many people across all countries of the world. However, such cellular therapies will be subject to the same challenges of immunological compatibility with the patient as currently experienced with solid organ and haematopoietic stem cell transplantation. Long-term immune suppression poses a number of risks for patients including general complications such as increased susceptibility to opportunistic infection, neoplasia and cardiovascular disease and specific side effects associated with individual immunosuppressive agents. In broad general terms, the closer the immunological matching between donor (and therefore the cellular product) and recipient, the lower the likely risk of rejection and degree of immunosuppression required. It is therefore advantageous to match as closely as possible for the major transplant antigen systems. The opportunity to pre-select donors for the generation of induced pluripotent stem cell (iPSC) lines opens up an opportunity not possible with human embryonic stem cell lines, which is to create a bank of cell lines specifically chosen to match the widest possible number of recipients worldwide. This will most efficiently be done by recruiting donors who are blood group O and are homozygous for common human leukocyte antigens (HLA). The diversity of HLA types means that it is highly unlikely that any single regional or national bank could contain sufficient cell lines to cover all people within their population base, and therefore, international collaboration between cell banks will be key to equity of access. This is very similar to the situation with haematopoietic stem cell registries and cord blood banks, whereby international collaboration enables access to a much larger pool of HLA-typed potential donors than that provided by individual countries. Such a global iPSC haplobank might provide a non-discriminatory platform for the development of iPSC-derived cellular therapeutics, but in practical terms, two conditions will need to be met: first, the data will have to demonstrate that the manufacturing process is reproducible and that cell lines derived from different donors can be generated to a similar standard; second, international regulators would need to accommodate the general proposition that a cellular product derived from one cell line as a starting material is sufficiently similar to that derived from another (subject to demonstration of comparability of biological characteristics) that it can be considered the same product for clinical and regulatory purposes—repeat clinical trials on patients of every different HLA type is clearly impracticable. This requires attention to the following issues:

## Immunological Composition of the GMP iPSC Haplobank

Transplantation of nucleated somatic cells, tissues and complex organs obtained from a randomly selected donor into an immune-competent host is almost invariably recognised and destroyed by the recipient immune system. There is, however, considerable variation in the survival time, and this reflects the degree of genetic disparity between the donor and recipient, correlating most closely with compatibility for ABO blood group and the level of matching for human leukocyte antigens (HLA). In clinical cell, tissue and organ transplantation, it is therefore desirable to match, as closely as possible, the blood group and HLA of the donor and recipient in order to reduce the immunogenic burden and minimise the rejection response.

The ideal scenario to achieve immunological compatibility of transplanted tissue is to use autologous cells and tissue obtained directly from the intended recipient, which is not therefore subject to immunological rejection. Clearly, this ideal also applies to transplantation of autologous iPSC-derived cellular therapies but to achieve this on a large scale would require the generation of individualised iPSC lines for many millions of potential beneficiaries worldwide which, at least for the time-being, is not a practicable proposition. A more practical approach to achieve HLA matching is to create a bank of HLA-typed iPSC lines from which cellular therapies can be manufactured which are immune compatible with the largest possible number of potential recipients [1].

HLA polymorphism has evolved from common ancestral genes and developed substantial heterogeneity in response to the enormous variety of environmental infectious agents and the potential for neoplastic transformation. As a result, HLA diversity is highest between populations and ethnic groups that have evolved in different geographical regions of the world. Despite such high levels of genetic diversity, certain HLA alleles and HLA haplotypes are conserved within populations, most likely through natural selection as a result of conferring a survival advantage to environmental challenges within a given geographical region. Therefore, the diversity of HLA polymorphism within populations and ethnic groups is lower, and some alleles and conserved haplotypes occur more frequently. For example, the HLA haplotype: HLA-A*01:01, HLA-B*08:01, HLA-C*07:02, HLA-DRB1*03:01, HLA-DRB3*01:01, HLA-DQA1*05:01 and HLA-DQB1*02:01 is carried by around 12–15 % of white northern Europeans and is also frequent amongst Americans of Hispanic and African ancestries [2, 3•]. In contrast other conserved HLA haplotypes are restricted to particular populations; for example, HLA-A24, HLA-B52, and HLA-DR15, and HLA-A11, HLA-B62, and HLA-DR4 haplotypes are present in around 20 % of the Japanese population but are much less common in northern Europeans [4, 5]. This observation led Turner et al. [6] and others [3•, 4, 7] to suggest that future iPSC banks should be populated using a relatively small number of lines that have common homozygous HLA haplotypes selected for maximum utility to match the intended recipient population (haplobank). Furthermore, because of the degree of sharing of conserved HLA haplotypes between ethnic groups, the formation of a global iPSC haplobank would be expected to reduce still further the total number of lines required to match most populations from different ancestral backgrounds and also those of mixed race [6].

Simulations of the estimated number of homozygous HLA lines required to provide HLA-A, HLA-B and HLA-DR compatible tissue in various populations worldwide have been undertaken for UK [4], Japanese [8], Chinese [9] and North Americans (of northern European, Hispanic, Asian and African ancestry) [3•], and all have shown that a surprisingly low number of HLA homozygous donors (between 50 and 150) would provide HLA-compatible tissue for around 50 to 90 % of the respective populations.

The identification of volunteers with the desired homozygous HLA types to donate tissue to populate a global iPSC haplobank would require the random screening of many hundreds of thousands of individuals collectively representing the broad range of human genetic diversity. An alternative and readily available source is to identify such individuals amongst the many millions of HLA-typed volunteers that are already registered worldwide on platelet apheresis panels, haematopoietic stem cell donor and cord blood registries, and healthy controls identified in genome-wide association studies. Furthermore, for homozygous HLA types in which there are many hundreds of potential donors, additional desirable criteria may also include selecting volunteer donors who are blood group O (as is done for *universal* blood donors) and female donors who carry two X-chromosomes and are compatible for minor HY histocompatibility loci. Attention will need to be paid to the practical, legal and ethical issues involved on approaching people who have volunteered to donate blood, cells or tissues for the treatment of individual patients rather than as a starting material for the manufacture of medicinal products. In addition, the nature of the informed consent given by those who choose to donate for this purpose needs to be considered, including the likely scope of testing for infectious agents and genetic abnormalities, the implications of long-term traceability and the circumstances under which they would be informed of findings of potential clinical significance, and the likelihood of commercial development of products derived from their donated material [10]. Whilst allogeneic cellular therapy products derived from such an iPSC haplobank are not likely going to be completely immune compatible with recipients, they may mitigate the degree of immune suppression required both in individuals and across the population as a whole. The alternative scenario suggests a substantial increase in the number of immune-suppressed individuals in the population as regenerative cellular therapies emerge into routine clinical practice.

Achieving the goal of making regenerative medicine available to a large and diverse population worldwide through the provision of a global iPSC haplobank will require extensive collaboration to ensure maximum utility and minimum redundancy of stored iPSC capable of meeting good manufacturing practice (GMP) requirements. Review of the HLA types present in populations around the world will allow determination of the optimal homozygous HLA panel required to match each and appropriate balancing to ensure that all ethnic groups are equitably represented.

## Donor Selection, Screening and Consent

Whilst a primary determinant of donor selection will be blood group and HLA type, a number of other considerations pertain including the general health of the donor her/himself and the risk of transmitting infection, neoplastic or genetic disease to the recipient(s). Routine donor selection and screening processes mitigate these risks in the context of clinical blood, tissue and organ transplantation, but it ought to be borne in mind that iPSC-derived therapies could be used for many patients receiving different kinds of cellular products over a prolonged period of time, at least some of whom are likely to be immunosuppressed. Consideration therefore needs to be given to extended screening of donors either generically or in the context of specific clinical applications.

Attention also needs to be given to the retention of traceability between donor(s) and recipient(s) for an extended period of time (potentially several decades and across international boundaries as required by Cell and Tissues Regulations in US and EU jurisdictions [11–13]). In addition, consideration should be given to the long-term follow up of recipients and the development of a registry with the capability to link potential patterns of short- and long-term adverse reaction arising in respect of a given cell line across geographic boundaries and different therapeutic applications.

Finally, agreement needs to be reached a propos the nature and extent of informed consent given the long-term retention of traceability and uncertainty over future screening and application of the iPSC lines—in particular the duty of care to the donor should findings arise which are of relevance to her/his health, family or public health.

Given the variation in the extent and nature of infectious and genetic risk, organisational infrastructure and cultural norms across the world, achieving consensus on even these donor-related issues may not be straightforward.

## GMP Manufacturing

The use of iPSC-derived cellular therapies in the clinic requires the products to be manufactured under good manufacturing practice (GMP). Globally, the regulations and guidelines for GMP vary to a certain extent depending on country and regulatory environment, but share the common objective of establishing minimum requirements to ensure that products are consistently produced and controlled to quality standards appropriate to their intended use.

The full manufacturing chain for a cell therapy product derived from iPSC requires good practices to be applied from the procurement of the donor tissue or cells, through the derivation and expansion of the iPSC line to the manufacture and characterisation of the cellular therapy product itself. In order to protect the health of both the donor and recipient(s), the procurement of starting cells or tissue for iPSC generation needs to comply with the standards set down in national law for blood, cell and tissue donation. A variety of approaches have been developed to effect cellular reprogramming. Commonality of approach or at least a common understanding of the strengths and weaknesses of different approaches would help prevent lack of comparability of different cell lines. Since iPSC lines can proliferate indefinitely in vitro, quality control measures need to be adequately designed to monitor genetic and epigenetic stability through derivation, seed lots and master cell banks over multiple passages. Appropriate control and testing will be crucial to ascertain that the iPSC line has not changed over prolonged storage. The ability of different iPSC lines to differentiate into different cell lineages in the context of standardised culture protocols will need to be assessed if the relative potency of different iPSC lines is to be properly understood. Finally, in addressing the risks from diverse sources of donation, more extensive evaluation of potential contamination with adventitious agents will be required which may require the application of new technologies. For these key scientific features, a common set of evaluation criteria and assays will be required to be applied to seed stocks of the candidate cell lines as discussed below.

A number of countries have embarked upon the manufacture of GMP iPSC lines, and several more countries are in the planning phase of such activities (pers comm. haplobank library survey). Manufacturing and up-scaling optimisation of iPSCs are currently global activities, and substantial dedicated public and private funding is invested in this area. As a consequence, many stakeholders are developing in-house GMP protocols and will protect these processes via a patent filing or trade secret strategy. Thus, the IP landscape is another factor to take into account when planning GMP manufacturing of iPSC lines since it is likely that there will be multiple patent holders independent of which manufacturing process is chosen. Several different manufacturing processes are likely to be acceptable to the community as long as an appropriate GMP standard is demonstrated. As discussed above, it is desirable if a minimum GMP standard could be agreed upon globally by the various international and national developers and regulatory bodies to ensure that, in due course, cell lines and relevant therapies can reach as large patient populations as possible in the future.

## Standardisation and Quality Control

Standardisation in stem cell research and development is clearly very challenging as a result of the complex cell biology and rapid development of the science and technologies involved.

Cultures of undifferentiated iPSC lines show a degree of variability in the phenotype of their undifferentiated state as they are passaged in vitro and also appear to respond differently when subjected to the same differentiation protocols [14•, 15]. Delivering large collections of tissue-type specific cell lines will require standardisation of minimum criteria and requirements for selection of the original cell sources.

Standardised approaches to culture and characterisation will be important to resolve the real differences between cell lines.

**Fig. 1:**
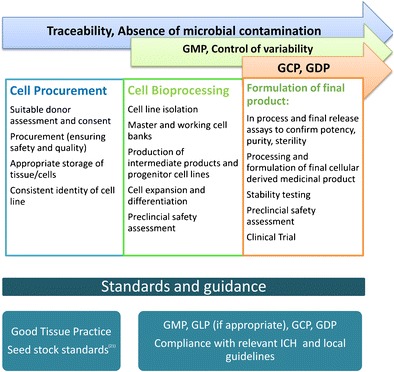
Progression of application of standards and guidance from donor material to cell therapy

Standards may be written documents providing general points to consider, guidance on technical procedures or even specific protocols required under certain regulatory requirements. They may also be physical preparations used to enable comparison of different cellular preparations [16]. Standardisation may be considered at three levels [17] (Fig. 1):

1. To confirm the key attributes of selected candidate cell sources and use a range of analytical techniques to compare them to enable final selection of the cell source of preference.
2. To establish standardised scale-up and analytical systems for the bioprocessing of the cells to make the therapeutic product including stable expansion of undifferentiated cells.
3. To establish standardised assays and reference materials to assess the key safety and efficacy properties of the final therapeutic product.

At this early stage of development, the standards of most value for iPSC banking for clinical use are the establishment of standards for preparation of seed stocks of early passage iPSC lines which will be based on five attributes as follows:

- Sterility (absence of microbial agents)
- Identity (correct origin)
- Purity (cellular composition)
- Potency (functional capability)
- Stability in vitro (changes that occur on passage or use in different culture environments)

### Sterility

All cell lines should be checked for the common laboratory contaminants. General hazards of bacterial and fungal contamination are screened for using protocols for sterility testing, although it is important to recognise that many species will not grow under the conditions of these tests. One of the commonest of these in cellular therapy culture is mycoplasma which can cause dramatic physiological and genetic changes in cell cultures and for which there are pharmacopoeial standard tests. For other, non-culturable organisms, routine microscopic inspection of culture may be the only means to identify them although 16S rRNA PCR may provide a useful screening method and may need to be used, as discussed above, for the diversity of cells used in haplobank next-generation technologies.

### Identity

Switching or cross-contamination of cell lines is a problem that has dogged the field of cell culture and could have a significant effect on cellular therapy development. In order to assure correct identity of iPSC lines, samples of the original tissue should be retained if possible, and DNA profiling (typically by STR genotyping) performed to show that the original tissue and derived cell line are from the same donor. Sharing of identity data amongst researchers and stem cell resource centres is desirable.

### Cellular Purity

Phenotypic identity is a complex issue due to the heterogenous populations often present in cell cultures. In addition, these change with time in culture. For undifferentiated pluripotent stem cells, there is some consensus on key phenotypic markers that should be used for banks of undifferentiated pluripotent stem cells [18•].

### Potency

This is an especially challenging aspect of iPSC characterisation, and the most fundamental aspect of this is the pluripotency of the cell culture which requires a defined set of protocols to show that cells can be directed into the three germline lineages required to generate all cells of the human body. Replication of such testing in different labs is very challenging and will be one of the early tests to require standardisation.

### Stability

As already mentioned, the stability of iPSC in culture is a significant concern for delivery of reproducible cells for therapy. Genomic instability could potentially give rise to transformed and potentially tumourigenic cells, which might persist in preparations of iPSC lines intended for patient therapy. It is unlikely that any cell culture is absolutely genetically stable, and stem cell lines, both iPSC and hESC, show genetic change in vitro culture as do other cell cultures [15]. An important issue is to be able to resolve the significance of observed changes [14•]. Low-level abnormal clones in a pluripotent cell lines are not unusual [16], and where mutations confer a growth advantage, they may rapidly dominate and replace all the original cells. Instability in the epigenetic profiles of iPSC lines is also prevalent [17]. It has been suggested that reprogramming can give rise to the abnormal expression of antigens not expressed (or expressed early) in normal development [19], or whether the in vitro culture process may give rise to abnormal lipid membrane structure or glycosylation patterns, which could lead to immune rejection [20]. The significance of such changes is yet to be determined and will only be answered through the examination of larger numbers of iPSC lines and their differentiated progeny.

An international consensus on the development of seed stocks of human pluripotent stem cell lines has been developed by the International Stem Cell Banking Initiative (ISCBI, in press) including contributors to the haplobank project.

## Regulatory Convergence and Harmonisation

A final problem in achieving common global acceptance of GMP iPSC lines is that even if the lines themselves are procured, manufactured and quality controlled in a similar manner, the regulatory environment varies between countries and may treat the lines and cellular therapy products derived therefrom differently. Part of the problem lies in the emergent nature of cell therapy from a background of blood, cell and tissue provision on the one hand and medicinal product manufacture on the other, the complex and rapidly evolving nature of the field and the need to build on and adapt regulatory legislation mainly designed for other purposes.

The challenge for the developers of such banks is to meet compliance with the legislation in each of the territories where a product from an internationally accessible haplobank may be used. Whilst there are international guidelines for the quality standards for biotechnology products, there are no such international guidelines for the sourcing of human donor cells; the method of manufacture, testing and quality standards for GMP compliant iPS banks; and subsequent preclinical and clinical testing and market authorisation approval of products derived from such haplobanks.

### Legislation

Using the three territories of the EU, US and Japan as examples, there are multiple legislative documents covering the selection of donor material, manufacture, testing and subsequent clinical use which need to be considered. In the EU, there are multinational legislative documents which cover the selection and eligibility of donors [11, 12], manufacture [21] and clinical use [22] of medicinal products with many associated guidance notes. Each of these directives is required to be translated in the national legislation of each EU member states. In the USA, the legislation is national and is defined in the Codes of Federal Regulation Title 21 [13], in particular 21 CFR Part 1271 (Donor Eligibility and Selection); Part 201 for Good Manufacturing Practices and Subchapters D (Drugs for Human Use) and F (Biologicals). In Japan, the products derived from a haplobank will be regulated under the Pharmaceutical Affairs Law [23] and its associated Guidelines, in particular the Guidelines on clinical research using human stem cells (2006).

### Donor Eligibility and Selection

Mandatory donor eligibility and selection criteria applicable in each territory aim to achieve the selection of human tissues and cells of the highest standard from donors who are free from, and pose a low risk of transmission of, relevant communicable diseases. There are many common requirements; however, there are also some differences which must be incorporated when developing the specification and consent for donors who will be recruited to contribute to the bank.

Likewise, although striving to achieve the same goal, there are divergent manufacturing standards; for example, there are different pharmacopoeial standards (USP, EP and JP), cleanroom operating, testing and traceability requirements. For example, there is a requirement to store donor-related records for 10 years in Japan and the US, whereas this is extended to 30 years in Europe. Furthermore, there is considerable divergence in the preclinical and clinical requirements of products derived from an induced pluripotent bank.

This environment therefore poses a significant challenge for the development of a global haplobank predicated on mutual recognition of iPSC lines and approval of the cellular therapies derived therefrom for clinical trial and market authorisation. Developers of haplobanks, and products derived from these, must take these divergent standards into consideration at an early stage in the development and establishment of the bank.

### Comparability of Products Derived From the Haplobank

The comparability of master cell banks (MCB) and cellular therapies derived from the various donors selected for the haplobank is arguably the key to the establishment of such a bank. A requirement for preclinical and clinical testing of each of the cell lines as if it were a separate product would negate the utility of a bank and restrict immunological matching of cellular therapies to a minority of potential recipients. The authors argue that, albeit that each iPSC line manufactured from different donors will be unique in some respects, they will each be manufactured and tested according to validated procedures and must meet the set in-process and iPSC line specification. As such, each cell line may be considered as a manufacturing lot of the haplobank. These lots in turn may form the starting material for any number of intermediate and final cell-based medicinal products, with each product type (e.g., iPSC-derived retinal pigment epithelial cells) having its own in-process and release specification. Assuming the extensive characterisation work performed on the MCB and downstream products demonstrates that the starting materials and subsequent clinical products are comparable, and within a set range of inter-batch variability, then it is reasonable to apply this approach in clinical trial. It is hence proposed that the haplobank cell lines could be used interchangeably to produce multiple lots of various products with the safety and efficacy of a product type derived from a number of different haplobank cell lines evaluated in one clinical trial. It is envisaged that a slightly larger number of subjects may be required but that all product lots should be viewed as comparable, and the data generated from each of these lots from the trial could be amalgamated. Early discussions with a number of major regulatory authorities have indicated the potential acceptability of this approach contingent upon the inclusion of adequate data in the dossier.

## Conclusions

As Bravery remarks in his insightful critique [24], the challenges in establishing a global GMP iPSC haplobank are formidable. Indeed, a cogent argument can be made that it is too early in the evolution of science to embark on such an ambitious endeavour. However, the counter-argument is that now is the time to attempt to reach international agreement around common standards for GMP iPSC manufacture before the field develops in a parochial manner circumscribed by national and regulatory boundaries [25, 26]. Recognition of the comparability of iPSC lines derived from different donors and manufacturers, and of the cellular therapies derived therefrom, would enhance the value of the donor’s gift and allow many more people access to life-enhancing or saving therapies in the future or reduce unnecessary exposure to high levels of immune suppression and the adverse consequences that brings. In light of these arguments, colleagues from centres around the world involved in the development of GMP-grade iPSC lines are working together to establish a Global Alliance for iPSC Therapies [6, 27•] aimed at harmonisation of standards [28] for donor selection and screening, iPSC manufacture, quality control and regulatory compliance as a prelude to establishment of a global GMP iPSC haplobank. Perhaps, ultimately, the argument for embarking on this path is predicated both on the principles of social justice and the requirements of commercial development.
